# U.S. charter schools neglect promoting physical activity: Content analysis of nationally representative elementary charter school websites

**DOI:** 10.1016/j.pmedr.2019.01.019

**Published:** 2019-02-07

**Authors:** David Kahan, Thomas L. McKenzie, Ashna Khatri

**Affiliations:** San Diego State University, School of Exercise and Nutritional Sciences, 5500 Campanile Drive, San Diego, CA 92182, USA

## Abstract

Most youths fail to meet recommended public health recommendations for physical activity (PA) and schools have been assigned a key role in mitigating this problem. Charter school growth exploded recently, but little is known about these schools' support of PA. School websites offer public windows through which they can share information about their programs, policies, and values. Thus, during spring 2018, we completed a quantitative content analysis of specific information about PA on the websites of a representative sample of U.S. charter elementary schools (*n* = 759). Nearly all schools (97%) had a functioning website, but most (52%) did not mention even one of five PA programs frequently offered at schools: physical education (PE), recess, intramurals, interscholastics, and PA clubs. PE, a standard part of school curricula, was mentioned on only 34% of the sites. Although public health proponents identify schools as ideal locations for promoting PA, school websites are neglected both as a means for identifying the importance of PE and PA and as a vehicle for informing students about how and when to be active during the school day.

## 1 Introduction

Physical activity (PA) is essential to growth and development of children and adolescents and their current and future health (2018 Physical Activity Guidelines Advisory Committee, 2018). As well, there is growing evidence that PA contributes to improved academic behavior and achievement (Centers for Disease Control and Prevention [CDC], 2010; Donnelly et al., 2016). Prominent health entities (Institute of Medicine [IOM], 2013; United States Department of Health and Human Services, 2008) recommend that children accrue at least 60 min of PA daily. Unfortunately, most children fall short of this target (Fakhouri et al., 2014; Troiano et al., 2008).

Schools are a key venues within the U.S. national strategy to increase PA (Centers for Disease Control and Prevention, 2013, Centers for Disease Control and Prevention, 2014; IOM, 2013; NPAPA, 2016; Rasberry et al., 2015), especially because they are attended by most children for extended periods and offer diverse PA opportunities (e.g. physical education (PE), recess, classroom breaks, before- and after-school programs). National bodies have suggested that half of children's recommended daily 60 MVPA minutes be accrued at school (CDC, 2013; IOM, 2013). To meet this goal, a whole-of-school approach, frequently referred to as Comprehensive School Physical Activity Programs (CSPAP), has been recommended (Centers for Disease Control and Prevention, 2013, Centers for Disease Control and Prevention, 2014; IOM, 2013; Rasberry et al., 2015).

CSPAPs involve collaborations among school personnel to promote and provide PA within diverse school contexts. Evidence suggests that having multiple school PA practices in place is related to increased student MVPA (Carlson et al., 2013); however, the overall adoption and effectiveness of CSPAPs are not yet well known. For example, the *School Health Profiles 2016* reported that only about 3% of U.S. secondary schools had a full CSPAP (Brener et al., 2017). Meanwhile, relative to their contributions to PA, the *2018 United States Report Card on Physical Activity for Children and Youth* has assigned schools a “D-” grade (NPAPA, 2018), an indication that schools are suceeding with <27% of students.

Studies of school PA policies and practices typically target public schools, but the *2018 Report Card* recommended private and charter schools be included in order to improve national surveillance (NPAPA, 2018). The U.S. political climate currently supports school choice, enabling children to bypass public schools in favor of charter and private school options (Bragg and Chingos, 2017; National Alliance for Public Charter Schools, 2018) Among these options, charter schools, which are public schools permitted to operate without abiding by many state and district regulations that apply to traditional public schools, have experienced tremendous growth since first being permitted by law in Minnesota in 1991.

Nationally representative data on school PA has almost exclusively relied on survey research, which has potential for recall error and social desirability bias. Meanwhile, school personnel sometimes refuse to participate in studies especially if the results might cast their school in a bad light (Thompson et al., 2018). A possibility for avoiding some of these complications is to use data posted on school websites—‘public windows’ through which schools share information about programs, policies, and values (Blackbaud, 2014). Charter schools also use websites in marketing and may include and exclude language and images to appeal to specific demographics (Wilson and Carlsen, 2016).

While studies of websites focusing on the provision and promotion of PA are rare and limited in scope and size, we recently conducted an analysis of PA-related content on the websites of 520 public elementary charter schools in California (Kahan and McKenzie, in press). Over a third of the websites (39%) did not mention the school provided any PA opportunities at all and only 31% mentioned a PE program—clear indications schools were not using the full potential of websites to inform constituents about PA or to promote it.

In 2017–2018, charter schools enrolled 3.2 million students in >7000 schools in the District of Columbia and 44 states, up from 1.2 million students in 4000 schools a decade earlier (David and Hesla, 2018). Little is known about how PA is represented on charter school websites across the country. Thus, we aimed to identify the prevalence of information related to PA on the websites of a representative sample of elementary charter schools in the US. Specifically, we were interested in information on the conduct of PE (frequency, lesson duration, volume, curriculum, PE teacher), recess (frequency, session duration, volume), and extracurricular PA programs (intramurals, interscholastic sports, PA clubs) and how it related to school demographic characteristics.

## 2 Methods

### 2.1 School selection

Supplementary material Fig. 1 illustrates steps taken to obtain our analytic sample of 759 schools. We first generated a list of U.S. charter schools by selecting the state and elementary school parameters from the *Find a Charter School* directory of the Center for Education Reform [CER] website. A priori, we generated 8 selection strata by coding schools by size [≥365 enrollment = 2 (large), ≤365 enrollment = 1 (small) based on the median enrollment of all elementary charter schools] and its city/neighborhood size [4 ≤ 100k, 3 = 100k–249k, 2 = 250k–999k, 1 ≥ 1 million based on values obtained from suburbanstats.org]. From this list, we initially excluded schools that were/had: 1) closed, 2) no working website, 3) pre-Kindergarten and Kindergarten only or combined elementary-through-high school grades, 4) virtual schools, 5) zero enrollment, and 6) duplicate listings.

Supplementary material Fig. 1 illustrates steps taken to obtain our analytic sample of 759 schools. We first generated a list of U.S. charter schools by selecting the state and elementary school parameters from the *Find a Charter School* directory of the Center for Education Reform [CER] website. A priori, we generated 8 selection strata by coding schools by size [≥365 enrollment = 2 (large), ≤365 enrollment = 1 (small) based on the median enrollment of all elementary charter schools] and its city/neighborhood size [4 ≤ 100k, 3 = 100k–249k, 2 = 250k–999k, 1 ≥ 1 million based on values obtained from suburbanstats.org]. From this list, we initially excluded schools that were/had: 1) closed, 2) no working website, 3) pre-Kindergarten and Kindergarten only or combined elementary-through-high school grades, 4) virtual schools, 5) zero enrollment, and 6) duplicate listings.

We used a sample size calculator (https://www.surveysystem.com/sscalc.htm), specifying a 95% confidence level and ±3% confidence interval, to generate the number of schools overall and then randomly sampled the derived number of schools for each stratum. During coding, we excluded 79 schools from the 8 strata, primarily because they had no website or were closed (see Supplementary material Fig. 1). For these, we randomly generated replacements to reflect their enrollment and city size. After coding the replacements, we excluded and replaced an additional 19 schools, resulting in an analytic sample of 759 schools.

### 2.2 Data/variable coding and treatment

We extracted data according to a predefined protocol (see Supplementary material 1) and created demographic variables in order to test relevant associations with the extracted information. As a proxy for socioeconomic status, we entered school zip codes into the United States Census Bureau's Fact Finder (https://factfinder.census.gov/faces/nav/jsf/pages/community_facts.xhtml#) app to derive median household income values based on the 2012–2016 American Community Survey 5-year estimates. We dichotomized those values as being above vs. below the median income of respective states (range across states, $40,528–$76,067) and categorized schools by grade level (i.e. elementary vs. elementary/middle) and enrollment (small vs. large).

We created additional variables from the webpage data. *PE mentioned* was coded 0 (no verbiage) or 1 (verbiage). When *PE frequency* was mentioned, we entered the average value (e.g. 2 to 3 days per week = 2.5) as a continuous variable. When *Lesson duration* was mentioned, we entered the number as a continuous variable. We calculated *PE volume* (min/week) as the product of PE frequency and duration.

We coded *PE teacher* existence, 0 (no teacher) or 1 (school had a PE teacher), *PE curriculum* presence, 0 (no verbiage), 1 (general verbiage such as locomotor skills, flexibility, endurance), and 2 (specific verbiage such as basketball drills, throwing mechanics), and *curriculum sequencing* 0 (no grade level sequencing) or 1 (sequencing across grades). We coded *recess mentioned*, 0 (no verbiage) or 1 (verbiage). For *recess frequency* we entered a single value from the website or an average value across grades. For *recess duration* we entered a single value from the website or an average value (when different across grades). We calculated *recess volume* (min/day) as the product of recess frequency and duration. We coded separately for *intramural sports*, *interscholastic* sports, and *PA clubs* 0 (no verbiage) or 1 (verbiage). Finally, we created a *total PA program opportunity* variable for each school to represent the sum of different PA program types mentioned on the website (i.e. PE, recess, intramural sport, interscholastic sport, and PA clubs; possible range = 0–5.)

### 2.3 Reliability analysis

Inter-rater reliability was assessed by comparing the results of a trained coder to those of the primary author on a random 5% sub-sample of schools. We calculated the prevalence index for each variable's cell array and used a threshold value of >│0.67│ to distinguish using kappa (κ) vs. prevalence-adjusted-bias-adjusted kappa (κ_*pb*_). The κ coefficients for two of the eleven variables ranged between (0.80–0.90) and κ_*pb*_ coefficients for the remaining nine variables ranged between (0.74–1.0).

### 2.4 Data analysis

We analyzed data with IBM SPSS Statistics 24 (Armonk, NY). We generated frequency and percentage values for all demographic and PE and PA characteristics across schools and generated measures of central tendency and variation for PE and recess frequency, duration, and volume data. Lastly, we conducted crosstabulations analyses between city size and school characteristics (i.e. SES, size, date opened, and grade levels) and PE and PA variables (i.e. *Mentioned PE*, *Mentioned PE Teacher*, and *≥1 Total PA Opportunity*). Significant chi-squared values were interpreted using effect size (Cramer's phi or V) and odds ratios with 95% confidence intervals.

## 3 Results

### 3.1 Charter school demographics

The 759 schools were from the District of Columbia and 39 of the 44 U.S. states that licensed charter schools. Roughly one-third were from three states (California, *n* = 130; Arizona, *n* = 62; and Florida, *n* = 57), with four states each represented by one school (Iowa, New Hampshire, Tennessee, Wyoming). Schools were primarily in cities with populations <100,000 (43.7%) and a majority (68.6%) were in areas where the median household income was below that for their respective state (Table 1). (We subsequently collapsed city population into three categories: <100,000 = small; 100,000–999,999 = medium; and ≥ 1 million = large). The numbers of elementary only and elementary/middle schools were similar (371 vs. 388), and most schools (65.5%) had fewer than 365 students (Table 1).

**Table 1:** Characteristics of randomly selected US charter schools teaching elementary grades (*n* = 759 schools): United States, 2018.

Characteristic	*f*	%
School location		
City population		
<100,000	332	43.7
100,000–249,999	92	12.1
250,000–999,999	191	25.2
≥1 million	144	19.0
Area neighborhood median income		
Below state median	521	68.6
Above state median	238	31.4

School characteristics		
Year school opened		
1993–2000	240	31.6
2001–2006	262	34.5
2007–2012	257	33.9
School type		
Elementary	371	48.9
Elementary/middle	388	51.1
School size		
Small (<365 students)	497	65.5
Large (≥365 students)	262	34.5

### 3.2 PA program opportunities

The proportions of schools with websites mentioning specific PA program opportunities was low: PE (34.1%), PA clubs (13.7%), interscholastic sports (9.1%), recess (7.9%), and intramurals (5.5%) (Table 2). In fact, 52.0% of the websites did not mention their school provided any PA program at all and no website mentioned providing all five PA program types (Table 2).

**Table 2:** Frequency and proportion of websites mentioning specific physical education (PE) and physical activity (PA) characteristics (*n* = 759 schools): United States, 2018.

Characteristic mentioned	*f*	%
PE	259	34.1
PE curriculum	54	7.1
Sequencing across grades	21	2.8
Specific content	15	2.0
PE teacher	284	37.4
PE specialist	46	6.1
Recess	60	7.9
Intramurals	42	5.5
Interscholastic sports	69	9.1
Physical activity clubs	104	13.7
Total PA program opportunities^a^		
0	395	52.0
1	230	30.3
2	106	14.0
3	20	2.6
4	8	1.1
5	0	0.0

a Sum of the different program types (PE, recess, intramurals, interscholastic sports, and PA clubs) mentioned.

Only 37.4% of the websites mentioned the school had a PE teacher and only 6.1% indicated the school had a teacher with specialist PE training (i.e. PE/kinesiology degree or PE license) (Table 2) Only 7.1% of sites mentioned a PE curriculum and only 2.8% and 2.0%, respectively, specified curriculum sequence or content.

Table 3 indicates few schools provided information on PE frequency and lesson length. Only 35 (4.6%) identified frequency (*M* = 3.3 ± 1.5 lessons/week) and only 21 (2.7%) mentioned lesson length (*M* = 43.0 ± 15.8 min/lesson). For the 16 schools (2.1%) providing sufficient information, the median PE volume was 135.0 ± 58.0 min/week (range = 60 to 420 min/week).

**Table 3:** Frequency, duration, and volume of physical education (PE) and recess reported on or calculated from websites (*n* = 759 total schools): United States, 2018.

Characteristic mentioned	*n*	*M* (*SD*) or *Med* (IQR)	Min	Max
PE				
Frequency (lessons/wk)	35	3.3 (1.5)	1.0	5.0
Lesson duration (min/lesson)	21	43.0 (15.8)	20.0	84.0
Volume (total min/wk)	16	135.0 (58.0)	60.0	420.0

Recess				
Frequency (sessions/day)	58	1.0 (0.0)	1.0	2.8
Recess duration (min/session)	57	19.0 (4.4)	10.0	30.0
Volume (total min/day)	57	20.0 (14.0)	10.0	60.0

Only 60 websites (7.9%) mentioned providing recess, with only 7.6% identifying its frequency (*Med* = 1.0 session/day) and 7.5% identifying its length (*M* = 19.0 ± 4.4 min/session) (Table 3). For the 57 websites mentioning both recess frequency and duration, the mean daily recess volume was 20.0 ± 14.0 min (range = 10 to 60 min).

### 3.3 Associations between school demographics and PA characteristics

Table 4 shows the associations between selected school characteristics (those in at least 33% of schools) and websites mentioning PE, PE teachers, and total school PA program opportunities. There were no significant associations between school age, school size, and school type (i.e., elementary vs. elementary/middle combination) and school websites mentioning PE, having a PE teacher, or offering ≥1 total PA program opportunities (see Table 4). Schools in small cities were more likely than those in medium (*OR* = 1.60, 95% CI = 1.15–2.23) and large (*OR* = 1.73, 95% CI = 1.14–2.61) cities to mention having a PE teacher. Additionally, schools in areas above their state's median income were more likely than those in lower-income areas to mention PE (*OR* = 1.74, 95% CI = 1.27–2.39), having a PE teacher (*OR* = 1.47, 95% CI = 1.07–2.01), and offering ≥1 total PA program opportunities (*OR* = 1.55, 95% CI = 1.14–2.11).

**Table 4:** Associations between school characteristics and websites mentioning of physical education (PE), PE teachers, and total physical activity (PA) program opportunities (*n* = 759 schools): United States, 2018.

Characteristic	Mentioned PE				Mentioned teacher				≥1 total PA opportunity			
	%	χ^2^	*p*	ES	%	χ^2^	*p*	ES	%	χ^2^	*p*	ES
Total	34.1				37.4				48.0			
City population		0.16	0.93			10.05	⁎⁎	0.12		0.28	0.87	
Small	33.7				44.0				47.0			
Medium	35.0				32.9				49.1			
Large	33.3				31.3				47.9			
Income		11.76	⁎⁎	0.12		5.84	⁎	0.09		7.82	⁎⁎	0.10
Below state median	30.1				34.5				44.5			
Above state median	42.9				43.7				55.5			
School age		2.53	0.31			2.71	0.26			2.34	0.31	
1993–2000	31.6				37.5				47.9			
2001–2006	34.5				40.8				44.7			
2007–2012	33.9				33.9				51.4			
School size		0.05	0.82			0.89	0.35			2.04	0.15	
Small	34.4				36.2				46.1			
Large	33.6				39.7				51.5			
Grade level		0.67	0.60			0.08	0.79			0.09	0.76	
Elementary	35.0				36.9				48.9			
Elementary/middle	33.2				37.9				51.1			

⁎ *p* < 0.05.

⁎⁎ *p* < 0.01.

## 4 Discussion

School choice legislation has brought about substantial increases in the establishment of public charter schools (David and Hesla, 2018). Public health authorities advocate schools provide diverse PA programs to enable students to accrue at least 30 of their total daily recommended 60 MVPA min on campus (CDC, 2013; IOM, 2013), but there has been little information on opportunities students have to participate in various CSPAP offerings in charter schools across the country.

The typical method for obtaining information on PE/PA policies and practices in large studies has been to use questionnaires, a procedure that can be burdensome to both investigators and school personnel and often produces low response rates (Chriqui et al., 2008). Questionnaires are also often completed by those only remotely involved with a school's PA program (e.g. district or state officials) and even when on-campus employees respond to questions, their answers may differ depending upon their position (e.g. school principal, PE teacher) (Lounsbery et al., 2013). A recent consensus statement from a CDC meeting of PA and measurement experts identified the need to explore accessing data from alternative sources (Fulton et al., 2016), and using the web has been suggested as a method to gather data from schools in a cost-effective manner (Kelly et al., 2017).

Most schools have public websites for sharing information about goals, programs, and staff, and in the case of charter schools, help to recruit students (Bragg and Chingos, 2017; Wilson and Carlsen, 2016). Understanding how websites contribute to PA and school-based PA offerings can provide useful insight for the improvement of school-based PA activities. Few studies of PA on school websites have been conducted (Kahan and McKenzie, in press), and the current investigation is the first to complete a quantitative analysis of content specifically related to PE and PA on the websites of a representative sample of U.S. elementary schools.

Nearly all (97%) schools initially selected for the study had a functioning public website. Nonetheless, the results clearly indicate that the websites were underutilized as means for informing constituents (e.g. enrolled students, families, and the community) about either the importance of PE and PA programs or when and how students might engage in them during the school day. For example, 52% of websites did not even mention their school provided even a single PA program and no website mentioned its school offered all five specific types (PE, recess, intramurals, interscholastics, club programs).

All U.S. states have mandates for PE (Kahan and McKenzie, 2017), and the Institute of Medicine (2013) has recommended an average daily PE dose of 30 min at the elementary school level. Of particular concern in the current study is that PE, the centerpiece of CSPAPs and the PA component most frequently identified in legislative policies, was mentioned on only 34% of the websites. Ostensibly there could have been just a large number of poor websites that neglected mentioning PE *and* academic subjects (e.g. English language arts [ELA], math). We therefore, a posteriori, examined a 10% random sample (*n* = 50) of school websites that omitted mention of PE. Of these schools’ websites: 68% each mentioned ELA and math; 44% and 32% mentioned specific content/specified a curriculum for ELA and math, respectively; and 24% and 18% provided ELA and math curriculum sequencing, respectively. Thus, we contend that relative to these two content areas, which are frequently subject to high-stakes testing, PE was far less prominently promoted on school websites. Unlike other PA programs in which participation is voluntary, PE has defined educational objectives (e.g. physical skills, physical fitness, knowledge acquisition, promotion of a physically active lifestyle) and is typically a standardized part of a school's curriculum (CDC, 2014). Even when mentioned, there was little information about PE being an important component of the school curriculum or detail on the content of classes or qualities of staff members teaching them. Less than 40% of websites mentioned the school had a PE teacher and only about 6% indicated the school employed a teacher with specialized PE training (i.e. PE/kinesiology degree, license, or certification). This is particularly germane because PE specialists typically function as the champions for other school PA programs and provide PE classes of higher quality than non-specialist teachers (CDC, 2014; IOM, 2013).

While the web has the potential for generating data from schools in a cost-effective manner (Kelly et al., 2017), the present study indicates that the quantity and quality of data on PA programs on the websites was inadequate for making widescale generalizations. For example, only 2% of schools reported sufficient information to permit the calculation of PE dosage, making it inappropriate to judge whether sample schools provided sufficient PE to meet nationally recommended standards (i.e. 150 min/week). Similarly, only 7.5% of schools reported recess volume, making it inappropriate to generalize as to whether schools were meeting national time recommendations for recess (i.e. 20 min/daily) (SHAPE America, 2016). As well, dosage data (e.g. number of students and how long they participated) for other PA programs were not provided, thus making it impossible to determine how well the schools stood relative to meeting the overall national recommendation of providing at least half of students' daily recommended 60 MVPA minutes. Subsequently, with the inadequacies of data currently on websites, researchers interested in how well schools are meeting national standards for PA programs still need to rely on using questionnaires with school and district personnel (Chriqui et al., 2008). Given the inadequacies of this method (Thompson et al., 2018), however, we recommend that studies also include on-site visits to schools in order to enhance and verify questionnaire findings. These visits should include the direct observation of PA programs and school records as well as interviews with personnel directly responsible for delivering the programs.

About two-thirds of the schools were in neighborhoods with median incomes below that for their state; these schools were less likely than those in wealthier areas to have websites mentioning they provided a PE program, had a PE teacher, and offered one or more PA program opportunities. Similar findings have been reported previously (Carlson et al., 2014), and these may reflect the ability of a school to offer certain programs as well as their intention to (or not) highlight them on their websites. Technical expertise is needed to develop and maintain websites, and perhaps schools in lower-income areas disproportionally allocate limited resources to areas other than PE and PA. Additionally, school programs need champions, and only 6% of the schools indicated employing a teacher with specialized PE training. Thus, it is possible that school personnel were unaware of the importance of PA/PE or how to promote and advocate for programs. On the other hand, school leaders in lower income areas may have considered PA/PE programs to be of limited importance to the school mission or assumed constituents were not interested in them. As school principals are the most influential people in establishing a school's goals and direction (ASCD and CDC, 2014), it is imperative they receive adequate training and incentives relative to them promoting PA engagement and other healthy practices at their schools.

### 4.1 Strengths and limitations

Study strengths include an analysis of the websites of 759 charter schools selected at random from across the U.S. using a template employed reliably by independent evaluators. Limitations include being unable to determine the currency of the information and the inability to visit schools to validate their website content. Additionally, the study was restricted to PA, PE, and sport variables and did not include other health-related content. We also assessed only website availability and content—not other features such as design, aesthetics, ease of use, or interactive capacity. Assessing website reach (e.g. number of hits) was not possible because those data were maintained by independent service providers.

### 4.2 Conclusion

Websites are part of nearly all U.S. schools and are a fundamental source for communicating important messages with students, parents, and staff. Our results show the websites of charter schools across the U.S. are rarely being used as a vehicle for advocating about PA or for promoting student engagement in on-campus PA programs. The lack of website recognition for PE as an important part of the school curriculum and the inattention given to it and other PA programs is an indication of their low status. A direct remedy is for PE/PA leaders to be formally trained in the use of digital marketing of their programs, from websites to social media. They should also press those responsible for school Web content to include specific information about their PE programs. Both recommendations are in keeping with components of SHAPE America (2017) beginning PE teacher Standard 6 (Professional responsibility). School principals are primarily influential in school matters, and in charter schools their decision-making latitude is expanded. Educating school principals about the importance of PA could possibly bring about changes. Nonetheless, making changes to PA programming, including the adoption of evidence-based PE curricula is highly influenced by external forces (Lounsbery et al., 2011). We recommend all schools assess their website content in order to ensure they are providing comprehensive and transparent messaging about PA and other health behaviors. We also recommend entities beyond the individual school, including state associations and the National Alliance for Public Charter Schools, become involved in advocating for PE and PA programs at schools.

The following are the supplementary data related to this article.

Supplementary Fig. 1 Flow diagram of national charter school population to analytic sample: United States, 2018. Supplementary Fig. 1

Supplementary material 1 Data extraction protocol and definitions, United States, 2018. Supplementary material 1
