# Supplementary material for: U.S. charter schools neglect promoting physical activity: Content analysis of nationally representative elementary charter school websites
Source: Prev Med Rep. 2019 Feb 7;14:100815. doi: 10.1016/j.pmedr.2019.01.019 (PMC6378835; doi:10.1016/j.pmedr.2019.01.019)
Supplement: Supplementary Fig. 1 — Flow diagram of national charter school population to analytic sample: United States, 2018. [file mmc1.pdf]

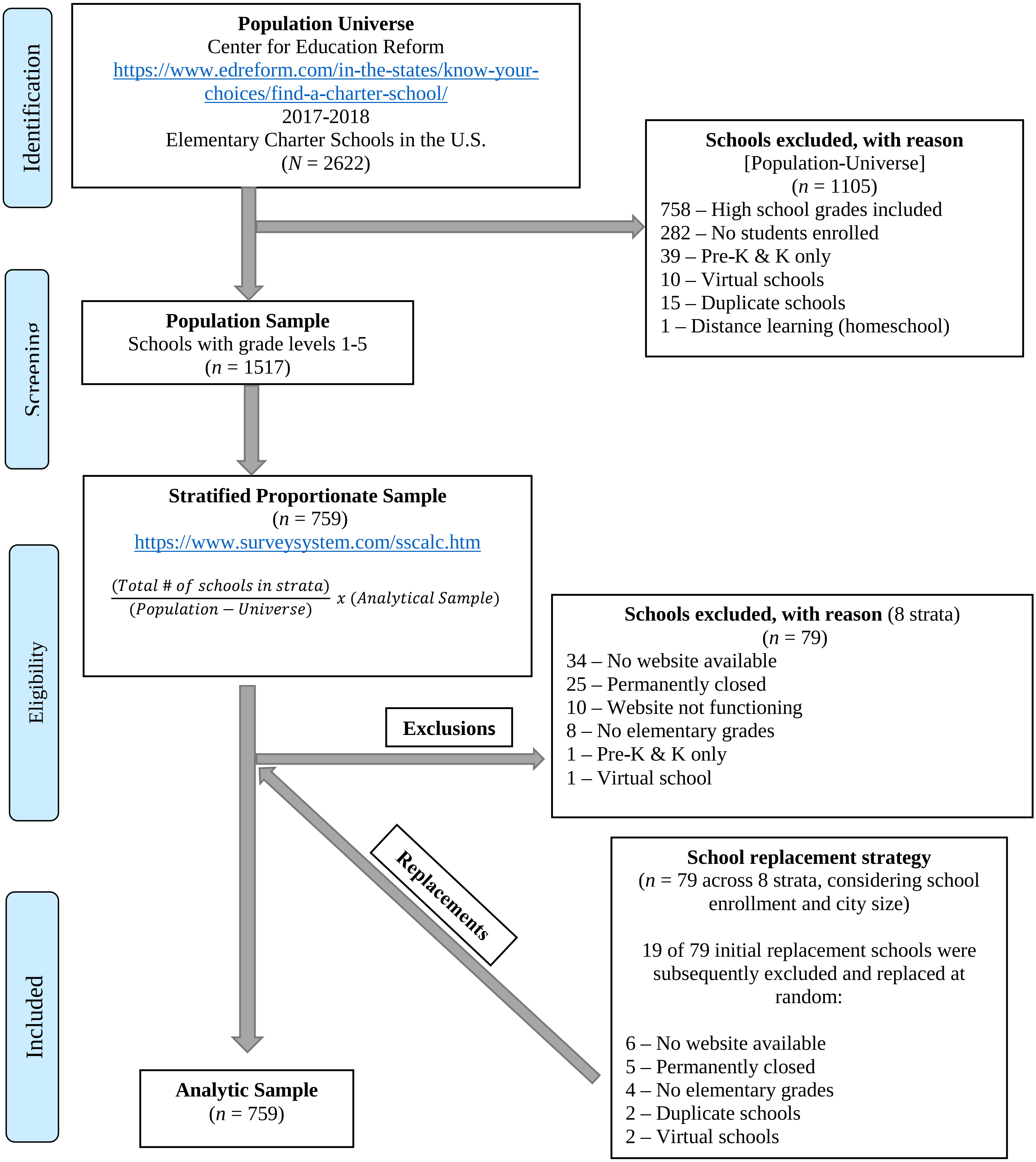
Identification
Screening
Eligibility
Included
Population Universe
Center for Education Reform
https://www.edreform.com/in-the-states/know-your-choices/find-a-charter-school/
2017-2018
Elementary Charter Schools in the U.S.
(N = 2622)
Schools excluded, with reason
[Population-Universe]
(n = 1105)
758 – High school grades included
282 – No students enrolled
39 – Pre-K & K only
10 – Virtual schools
15 – Duplicate schools
1 – Distance learning (homeschool)
Population Sample
Schools with grade levels 1-5
(n = 1517)
Stratified Proportionate Sample
(n = 759)
https://www.surveysystem.com/sscalc.htm
(Total # of schools in strata) / (Population – Universe) x (Analytical Sample)
Exclusions
Schools excluded, with reason (8 strata)
(n = 79)
34 – No website available
25 – Permanently closed
10 – Website not functioning
8 – No elementary grades
1 – Pre-K & K only
1 – Virtual school
Replacements
School replacement strategy
(n = 79 across 8 strata, considering school enrollment and city size)
19 of 79 initial replacement schools were subsequently excluded and replaced at random:
6 – No website available
5 – Permanently closed
4 – No elementary grades
2 – Duplicate schools
2 – Virtual schools
Analytic Sample
(n = 759)

Supplementary Fig 1.
Flow diagram of national charter school population to analytic sample: United States, 2018.
