# Supplementary material for: U.S. charter schools neglect promoting physical activity: Content analysis of nationally representative elementary charter school websites
Source: Prev Med Rep. 2019 Feb 7;14:100815. doi: 10.1016/j.pmedr.2019.01.019 (PMC6378835; doi:10.1016/j.pmedr.2019.01.019)
Supplement: Supplementary material 1 — Data extraction protocol and definitions, United States, 2018. [file mmc2.docx]

Supplementary Material 1. Data extraction protocol and definitions, United States, 2018.

______________________________________________________________________________

For schools meeting inclusionary criteria, we recorded the following from the original CER 2017-2018 academic school year directory: school name and website hyperlink, zip code, school size, open date, and grade level (K-6 = elementary, K-8 = elementary/middle). We used the ‘Command F’ or ‘ctrl F’ function to search key words such as *physical education*, *PE*, and *recess* across a school website. We also searched pages/links/tabs typically labeled “About Us,” “Academics/Programs,” “Bell Schedule,” “Athletics,” and “Campus/Student Life,” and their respective dropdowns.

From each school’s website, we recorded/calculated the following:

1) mention of “PE,” “physical education,” or “games” (for Waldorf schools) anywhere on the website, except when only identifying a specific PE teacher;

2) mention of frequency (lessons/week) and duration (min/lesson) of PE based on verbiage identifying their occurrence either generally (eg, “regularly,” “every week”) or specifically (e.g. “twice weekly,” “daily”);

3) mention of PE curriculum and curriculum sequence based on verbiage identifying the curriculum scope of PE either generally (e.g. fitness activities, games, locomotor skills) or specifically (e.g. basketball, soccer, or an elaboration provided on the specificity of the curriculum) and its sequence (e.g. grade by grade; lower vs. upper elementary);

4) mention of a PE teacher by surname and whether the person was a specialist (i.e. state credential; had an undergraduate or graduate degree in PE or Kinesiology);

5) mention of “recess” anywhere on website;

6) mention of frequency (periods/day) and duration (min/period) of recess (when combined with lunch on the bell schedule, we allocated half the time to recess);

7) mention of intramural sports based on verbiage indicating the school provided at least one intramural sport opportunity or team was available for students in at least one elementary grade level;

8) mention of interscholastic sports based on verbiage indicating at least one interscholastic sport opportunity (stated explicitly or game day schedule and league affiliation was provided) was provided for students in at least one elementary school grade level; and

9) mention of a PA club (e.g. dance, running club, soccer, yoga, karate) provided by the school or outside source to students in at least one elementary school grade level.

______________________________________________________________________________
